# Diaqua­bis­(4-bromo­benzoato-κ*O*)bis­(*N*,*N*-diethyl­nicotinamide-κ*N* ^1^)manganese(II)

**DOI:** 10.1107/S1600536811031412

**Published:** 2011-08-06

**Authors:** Hacali Necefoğlu, Füreya Elif Özbek, Vijdan Öztürk, Vedat Adıgüzel, Tuncer Hökelek

**Affiliations:** a Kafkas University, Department of Chemistry, 36100 Kars, Turkey; b Hacettepe University, Department of Physics, 06800 Beytepe, Ankara, Turkey

## Abstract

In the crystal structure of the title Mn^II^ complex, [Mn(C_7_H_4_BrO_2_)_2_(C_10_H_14_N_2_O)_2_(H_2_O)_2_], the Mn^II^ cation is located on an inversion center and coordinated by two diethyl­nicotinamide (DENA) ligands, two 4-bromo­benzoate (PBB) anions and two water mol­ecules in a distorted octa­hedral geometry. The dihedral angle between the carboxyl­ate group and the adjacent benzene ring is 3.25 (14)°. In the mol­ecule, the pyridine ring and the benzene ring are oriented at a dihedral angle of 77.24 (5)°. In the crystal, inter­molecular C—H⋯O hydrogen bonds link the mol­ecules into a two-dimensional network. Weak inter­molecular C—H⋯O hydrogen bonds and π–π inter­actions between the pyridine rings of neighbouring mol­ecules [centroid–centroid distance = 3.537 (1) Å] further consolidate the crystal packing.

## Related literature

For literature on niacin, see: Krishnamachari (1974 ▶). For information on the nicotinic acid derivative *N*,*N*-diethyl­nicotinamide, see: Bigoli *et al.* (1972 ▶). For related structures, see: Hökelek *et al.* (1996 ▶, 2009*a* ▶,*b* ▶); Hökelek & Necefoğlu (1998 ▶, 2007 ▶); Necefoğlu *et al.* (2011 ▶). For bond-length data, see: Allen *et al.* (1987 ▶).
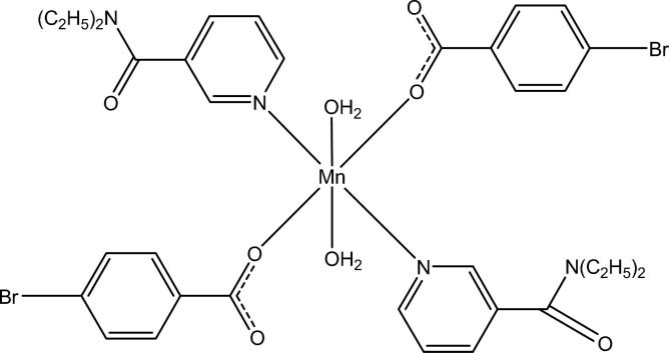


## Experimental

### 

#### Crystal data

[Mn(C_7_H_4_BrO_2_)_2_(C_10_H_14_N_2_O)_2_(H_2_O)_2_]
*M* *_r_* = 847.46
Triclinic, 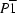

*a* = 7.2939 (2) Å
*b* = 8.5130 (2) Å
*c* = 16.1252 (4) Å
α = 83.970 (3)°
β = 79.529 (3)°
γ = 68.031 (2)°
*V* = 912.34 (4) Å^3^
*Z* = 1
Mo *K*α radiation
μ = 2.61 mm^−1^
*T* = 100 K
0.35 × 0.25 × 0.20 mm

#### Data collection

Bruker Kappa APEXII CCD area-detector diffractometer
Absorption correction: multi-scan (*SADABS*; Bruker, 2005 ▶) *T*_min_ = 0.462, *T*_max_ = 0.594
16194 measured reflections
4616 independent reflections
4127 reflections with *I* > 2σ(*I*)
*R*_int_ = 0.029

#### Refinement

*R*[*F* ^2^ > 2σ(*F* ^2^)] = 0.026
*wR*(*F* ^2^) = 0.073
*S* = 1.09
4616 reflections
233 parameters
2 restraints
H atoms treated by a mixture of independent and constrained refinement
Δρ_max_ = 0.42 e Å^−3^
Δρ_min_ = −0.39 e Å^−3^

### 

Data collection: *APEX2* (Bruker, 2007 ▶); cell refinement: *SAINT* (Bruker, 2007 ▶); data reduction: *SAINT*; program(s) used to solve structure: *SHELXS97* (Sheldrick, 2008 ▶); program(s) used to refine structure: *SHELXL97* (Sheldrick, 2008 ▶); molecular graphics: *ORTEP-3 for Windows* (Farrugia, 1997 ▶); software used to prepare material for publication: *WinGX* (Farrugia, 1999 ▶) and *PLATON* (Spek, 2009 ▶).

## Supplementary Material

Crystal structure: contains datablock(s) I, global. DOI: 10.1107/S1600536811031412/xu5285sup1.cif

Structure factors: contains datablock(s) I. DOI: 10.1107/S1600536811031412/xu5285Isup2.hkl

Additional supplementary materials:  crystallographic information; 3D view; checkCIF report

## Figures and Tables

**Table 1:** Hydrogen-bond geometry (Å, °)

*D*—H⋯*A*	*D*—H	H⋯*A*	*D*⋯*A*	*D*—H⋯*A*
O4—H41⋯O2	0.86 (3)	1.82 (3)	2.6606 (18)	165 (3)
O4—H42⋯O3	0.82 (2)	1.92 (3)	2.742 (2)	166 (3)
C6—H6⋯O2^i^	0.93	2.30	3.168 (3)	155
C10—H10⋯O2	0.93	2.44	3.353 (2)	168

Symmetry code: (i) 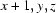.

**Fig. 1.**
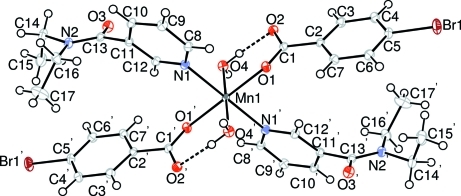
The molecular structure of the title molecule with the atom-numbering scheme. Displacement ellipsoids are drawn at the 50% probability level [symmetry code ('): -x, -y, -z]. Hydrogen bonds are shown as dashed lines.

**Fig. 2.**
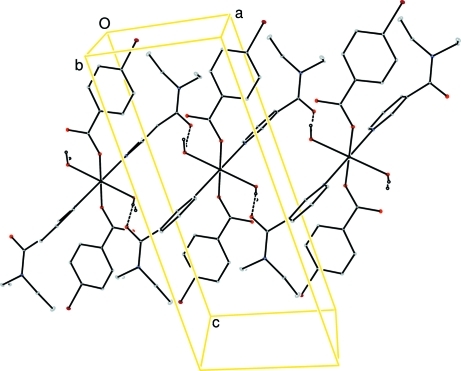
A view of the crystal packing of the title compound. Only the intermolecular O—H···O hydrogen bonds are shown as dashed lines. [H-atoms not involved in hydrogen bonding have been omitted for clarity].

## supplementary crystallographic information

### Comment

As a part of our ongoing investigations of transition metal complexes of nicotinamide (NA), one form of niacin (Krishnamachari, 1974), and/or the nicotinic acid derivative *N*,*N*-diethylnicotinamide (DENA), an important respiratory stimulant (Bigoli *et al.*, 1972), the title compound was synthesized and its crystal structure is reported herein.

The asymmetric unit of the title mononuclear Mn^II^ complex, (Fig. 1), contains one-half molecule, the Mn^II^ atom being located on an inversion center. It consists of two *N*,*N*-diethylnicotinamide (DENA), two 4-bromobenzoate (PBB) ligands and two coordinated water molecules, all ligands coordinating in a monodentate manner. The crystal structures of similar omplexes of Cu^II^, Co^II^, Ni^II^, Mn^II^ and Zn^II^ ions, [Cu(C_7_H_5_O_2_)_2_(C_10_H_14_N_2_O)_2_] (Hökelek *et al.*, 1996), [Co(C_6_H_6_N_2_O)_2_(C_7_H_4_NO_4_)_2_(H_2_O)_2_] (Hökelek & Necefoğlu, 1998), [Co(C_9_H_9_O_2_)_2_(C_10_H_14_N_2_O)_2_(H_2_O)_2_] (Necefoğlu *et al.*, 2011), [Ni(C_7_H_4_ClO_2_)_2_(C_6_H_6_N_2_O)_2_(H_2_O)_2_] (Hökelek *et al.*, 2009*a*), [Mn(C_9_H_10_NO_2_)_2_(H_2_O)_4_].2H_2_O (Hökelek & Necefoğlu, 2007) and [Zn(C_7_H_4_BrO_2_)_2_(C_6_H_6_N_2_O)_2_(H_2_O)_2_] (Hökelek *et al.*, 2009*b*) have also been reported. In the copper(II) complex mentioned above the two benzoate ions coordinate to the Cu^II^ atom as bidentate ligands, while in the other structures all the ligands coordinate in a monodentate manner.

In the title complex,  the four symmetry related O atoms (O1, O1', O4 and O4') in the equatorial plane around the Mn^II^ ion form a slightly distorted square-planar arrangement, while the slightly distorted octahedral coordination is completed by the two N atoms of the DENA ligands (N1 and N1') in the axial positions. The intramolecular O—H···O hydrogen bonds (Table 1, Fig. 1) link the water molecules to the carboxylate oxygens. The near equalities of the C1—O1 [1.266 (2) Å] and C1—O2 [1.256 (2) Å] bonds in the carboxylate groups indicate delocalized bonding arrangements, rather than localized single and double bonds. The Mn—O bond lengths are 2.1542 (12) Å (for benzoate oxygen) and 2.2088 (13) Å (for water oxygen), and the Mn—N bond length is 2.2632 (14) Å, close to standard values (Allen *et al.*, 1987). The Mn atom is displaced out of the mean-plane of the carboxylate group (O1/C1/O2) by 0.9729 (1) Å. The dihedral angle between the planar carboxylate group and the adjacent benzene ring A (C2—C7) is 3.25 (14)°. The benzene A (C2—C7) and the pyridine B (N1/C8—C12) rings are oriented at a dihedral angle of A/B = 77.24 (5)°.

In the crystal, intermolecular O—H···O hydrogen bonds (Table 1, Fig. 2) link the molecules into a two-dimensional network. Weak intermolecular C—H···O hydrogen bonds (Table 1) and π···π interactions between the pyridine rings, Cg2—Cg2^i^, of neighbouring molecules [centroid-centroid distance = 3.537 (1) Å; symmetry code: (i) -x, 1 - y, 1 - z; Cg2 is the centroid of the ring B (N1/C8—C12)] further consolidate the crystal packing.

### Experimental

The title compound was prepared by the reaction of MnSO_4_.H_2_O (0.85 g, 5 mmol) in H_2_O (25 ml) and DENA (1.78 g, 10 mmol) in H_2_O (25 ml) with sodium 4-bromobenzoate (2.23 g, 10 mmol) in H_2_O (100 ml) at room temperature. The mixture was filtered and set aside to crystallize at ambient temperature for two weeks, giving colorless single crystals.

### Refinement

Atoms H41 and H42 (for water molecules) were located in a difference Fourier map and were freely refined. The C-bound H-atoms were positioned geometrically with C—H = 0.93, 0.97 and 0.96 Å, for aromatic, methylene and methyl H-atoms, respectively, and constrained to ride on their parent atoms, with *U*_iso_(H) = k × *U*_eq_(C), where k = 1.5 for methyl H-atoms and k = 1.2 for all other H-atoms.

### Crystal data

**Table:**

[Mn(C_7_H_4_BrO_2_)_2_(C_10_H_14_N_2_O)_2_(H_2_O)_2_]	*Z* = 1
*M**_r_* = 847.46	*F*(000) = 431
Triclinic, *P*1	*D*_x_ = 1.542 Mg m^−^^3^
Hall symbol: -P 1	Mo *K*α radiation, λ = 0.71073 Å
*a* = 7.2939 (2) Å	Cell parameters from 9256 reflections
*b* = 8.5130 (2) Å	θ = 2.6–28.5°
*c* = 16.1252 (4) Å	µ = 2.61 mm^−^^1^
α = 83.970 (3)°	*T* = 100 K
β = 79.529 (3)°	Block, colorless
γ = 68.031 (2)°	0.35 × 0.25 × 0.20 mm
*V* = 912.34 (4) Å^3^	

### Data collection

**Table:**

Bruker Kappa APEXII CCD area-detector diffractometer	4616 independent reflections
Radiation source: fine-focus sealed tube	4127 reflections with *I* > 2σ(*I*)
graphite	*R*_int_ = 0.029
φ and ω scans	θ_max_ = 28.7°, θ_min_ = 2.6°
Absorption correction: multi-scan (*SADABS*; Bruker, 2005)	*h* = −9→8
*T*_min_ = 0.462, *T*_max_ = 0.594	*k* = −11→11
16194 measured reflections	*l* = −21→21

### Refinement

**Table:**

Refinement on *F*^2^	Primary atom site location: structure-invariant direct methods
Least-squares matrix: full	Secondary atom site location: difference Fourier map
*R*[*F*^2^ > 2σ(*F*^2^)] = 0.026	Hydrogen site location: inferred from neighbouring sites
*wR*(*F*^2^) = 0.073	H atoms treated by a mixture of independent and constrained refinement
*S* = 1.09	*w* = 1/[σ^2^(*F*_o_^2^) + (0.0308*P*)^2^ + 0.6418*P*] where *P* = (*F*_o_^2^ + 2*F*_c_^2^)/3
4616 reflections	(Δ/σ)_max_ = 0.001
233 parameters	Δρ_max_ = 0.42 e Å^−^^3^
2 restraints	Δρ_min_ = −0.39 e Å^−^^3^

### Special details

**Table:**

Geometry. All esds (except the esd in the dihedral angle between two l.s. planes) are estimated using the full covariance matrix. The cell esds are taken into account individually in the estimation of esds in distances, angles and torsion angles; correlations between esds in cell parameters are only used when they are defined by crystal symmetry. An approximate (isotropic) treatment of cell esds is used for estimating esds involving l.s. planes.
Refinement. Refinement of F^2^ against ALL reflections. The weighted R-factor wR and goodness of fit S are based on F^2^, conventional R-factors R are based on F, with F set to zero for negative F^2^. The threshold expression of F^2^ > 2sigma(F^2^) is used only for calculating R-factors(gt) etc. and is not relevant to the choice of reflections for refinement. R-factors based on F^2^ are statistically about twice as large as those based on F, and R- factors based on ALL data will be even larger.

### Fractional atomic coordinates and isotropic or equivalent isotropic displacement parameters (Å^2^)

**Table:**

	*x*	*y*	*z*	*U*_iso_*/*U*_eq_	
Br1	−0.75487 (3)	−0.83337 (3)	0.028844 (11)	0.02549 (7)	
Mn1	−1.5000	−1.0000	0.5000	0.01072 (8)	
O1	−1.38994 (18)	−0.87032 (15)	0.39214 (7)	0.0149 (2)	
O2	−1.58179 (18)	−0.86624 (16)	0.29746 (8)	0.0162 (2)	
O3	−2.33701 (19)	−0.67456 (16)	0.62449 (8)	0.0180 (3)	
O4	−1.73146 (19)	−1.01296 (16)	0.43251 (8)	0.0164 (2)	
H41	−1.695 (5)	−0.972 (4)	0.3842 (14)	0.061 (10)*	
H42	−1.724 (4)	−1.107 (2)	0.4215 (17)	0.039 (7)*	
N1	−1.7301 (2)	−0.75479 (18)	0.55238 (9)	0.0135 (3)	
N2	−2.3337 (2)	−0.57656 (18)	0.74841 (9)	0.0154 (3)	
C1	−1.4231 (3)	−0.8666 (2)	0.31747 (10)	0.0132 (3)	
C2	−1.2580 (3)	−0.8627 (2)	0.24697 (10)	0.0122 (3)	
C3	−1.2803 (3)	−0.8670 (2)	0.16323 (11)	0.0156 (3)	
H3	−1.3960	−0.8757	0.1511	0.019*	
C4	−1.1308 (3)	−0.8583 (2)	0.09777 (11)	0.0167 (3)	
H4	−1.1453	−0.8606	0.0418	0.020*	
C5	−0.9600 (3)	−0.8463 (2)	0.11768 (11)	0.0159 (3)	
C6	−0.9306 (3)	−0.8470 (2)	0.20023 (11)	0.0158 (3)	
H6	−0.8128	−0.8420	0.2121	0.019*	
C7	−1.0816 (3)	−0.8554 (2)	0.26481 (10)	0.0145 (3)	
H7	−1.0647	−0.8561	0.3207	0.017*	
C8	−1.7011 (3)	−0.6072 (2)	0.54066 (10)	0.0142 (3)	
H8	−1.5813	−0.6055	0.5095	0.017*	
C9	−1.8416 (3)	−0.4569 (2)	0.57292 (11)	0.0158 (3)	
H9	−1.8164	−0.3567	0.5635	0.019*	
C10	−2.0201 (3)	−0.4589 (2)	0.61946 (11)	0.0148 (3)	
H10	−2.1162	−0.3603	0.6426	0.018*	
C11	−2.0531 (3)	−0.6112 (2)	0.63100 (10)	0.0131 (3)	
C12	−1.9050 (3)	−0.7549 (2)	0.59574 (10)	0.0137 (3)	
H12	−1.9281	−0.8561	0.6025	0.016*	
C13	−2.2514 (3)	−0.6235 (2)	0.66923 (11)	0.0138 (3)	
C14	−2.5372 (3)	−0.5766 (2)	0.77833 (12)	0.0203 (4)	
H14A	−2.6183	−0.5272	0.7341	0.024*	
H14B	−2.5964	−0.5054	0.8266	0.024*	
C15	−2.5430 (3)	−0.7523 (2)	0.80336 (13)	0.0261 (4)	
H15A	−2.6787	−0.7433	0.8232	0.039*	
H15B	−2.4636	−0.8021	0.8474	0.039*	
H15C	−2.4902	−0.8223	0.7553	0.039*	
C16	−2.2354 (3)	−0.5264 (2)	0.80695 (11)	0.0192 (4)	
H16A	−2.3176	−0.4126	0.8246	0.023*	
H16B	−2.1082	−0.5237	0.7775	0.023*	
C17	−2.1988 (4)	−0.6441 (3)	0.88473 (14)	0.0370 (5)	
H17A	−2.1306	−0.6066	0.9195	0.056*	
H17B	−2.1182	−0.7572	0.8678	0.056*	
H17C	−2.3246	−0.6427	0.9160	0.056*	

### Atomic displacement parameters (Å^2^)

**Table:**

	*U*^11^	*U*^22^	*U*^33^	*U*^12^	*U*^13^	*U*^23^
Br1	0.02017 (11)	0.04066 (12)	0.01687 (10)	−0.01507 (9)	0.00437 (7)	−0.00377 (8)
Mn1	0.00970 (17)	0.01062 (15)	0.01099 (16)	−0.00281 (13)	−0.00032 (13)	−0.00241 (12)
O1	0.0165 (6)	0.0158 (6)	0.0132 (5)	−0.0070 (5)	−0.0006 (5)	−0.0021 (4)
O2	0.0120 (6)	0.0194 (6)	0.0172 (6)	−0.0056 (5)	−0.0026 (5)	−0.0003 (5)
O3	0.0163 (6)	0.0198 (6)	0.0203 (6)	−0.0083 (5)	−0.0027 (5)	−0.0043 (5)
O4	0.0175 (6)	0.0174 (6)	0.0167 (6)	−0.0085 (5)	−0.0031 (5)	−0.0022 (5)
N1	0.0128 (7)	0.0138 (6)	0.0136 (6)	−0.0042 (5)	−0.0019 (5)	−0.0025 (5)
N2	0.0155 (7)	0.0146 (6)	0.0152 (7)	−0.0055 (6)	0.0013 (6)	−0.0024 (5)
C1	0.0151 (8)	0.0084 (7)	0.0138 (7)	−0.0024 (6)	0.0003 (6)	−0.0022 (5)
C2	0.0121 (8)	0.0104 (7)	0.0130 (7)	−0.0026 (6)	−0.0011 (6)	−0.0021 (6)
C3	0.0140 (8)	0.0174 (8)	0.0158 (8)	−0.0056 (7)	−0.0024 (6)	−0.0022 (6)
C4	0.0183 (9)	0.0187 (8)	0.0124 (7)	−0.0057 (7)	−0.0014 (7)	−0.0030 (6)
C5	0.0142 (8)	0.0176 (8)	0.0151 (8)	−0.0061 (7)	0.0018 (6)	−0.0023 (6)
C6	0.0126 (8)	0.0178 (8)	0.0181 (8)	−0.0065 (7)	−0.0031 (6)	−0.0009 (6)
C7	0.0179 (9)	0.0138 (7)	0.0125 (7)	−0.0062 (7)	−0.0022 (6)	−0.0019 (6)
C8	0.0126 (8)	0.0157 (7)	0.0150 (7)	−0.0063 (6)	−0.0014 (6)	−0.0011 (6)
C9	0.0188 (9)	0.0133 (7)	0.0172 (8)	−0.0076 (7)	−0.0028 (7)	−0.0017 (6)
C10	0.0158 (8)	0.0112 (7)	0.0155 (7)	−0.0026 (6)	−0.0009 (6)	−0.0040 (6)
C11	0.0129 (8)	0.0154 (7)	0.0114 (7)	−0.0053 (6)	−0.0019 (6)	−0.0020 (6)
C12	0.0141 (8)	0.0120 (7)	0.0150 (7)	−0.0048 (6)	−0.0019 (6)	−0.0014 (6)
C13	0.0132 (8)	0.0095 (7)	0.0165 (8)	−0.0021 (6)	−0.0008 (6)	−0.0016 (6)
C14	0.0173 (9)	0.0177 (8)	0.0233 (9)	−0.0068 (7)	0.0066 (7)	−0.0053 (7)
C15	0.0253 (10)	0.0219 (9)	0.0304 (10)	−0.0119 (8)	0.0064 (8)	−0.0034 (8)
C16	0.0248 (10)	0.0197 (8)	0.0146 (8)	−0.0098 (7)	−0.0015 (7)	−0.0034 (6)
C17	0.0505 (15)	0.0392 (12)	0.0281 (11)	−0.0209 (11)	−0.0191 (11)	0.0106 (9)

### Geometric parameters (Å, °)

**Table:**

Br1—C5	1.9009 (17)	C6—H6	0.9300
Mn1—O1	2.1542 (12)	C7—C2	1.393 (2)
Mn1—O1^i^	2.1542 (12)	C7—H7	0.9300
Mn1—O4	2.2088 (13)	C8—C9	1.387 (2)
Mn1—O4^i^	2.2089 (13)	C8—H8	0.9300
Mn1—N1	2.2632 (14)	C9—H9	0.9300
Mn1—N1^i^	2.2632 (14)	C10—C9	1.385 (2)
O1—C1	1.266 (2)	C10—C11	1.393 (2)
O2—C1	1.256 (2)	C10—H10	0.9300
O3—C13	1.238 (2)	C11—C13	1.503 (2)
O4—H41	0.860 (18)	C12—C11	1.386 (2)
O4—H42	0.823 (17)	C12—H12	0.9300
N1—C8	1.339 (2)	C14—C15	1.521 (2)
N1—C12	1.339 (2)	C14—H14A	0.9700
N2—C13	1.341 (2)	C14—H14B	0.9700
N2—C14	1.475 (2)	C15—H15A	0.9600
N2—C16	1.465 (2)	C15—H15B	0.9600
C1—C2	1.506 (2)	C15—H15C	0.9600
C3—C2	1.395 (2)	C16—C17	1.518 (3)
C3—H3	0.9300	C16—H16A	0.9700
C4—C3	1.391 (2)	C16—H16B	0.9700
C4—C5	1.383 (3)	C17—H17A	0.9600
C4—H4	0.9300	C17—H17B	0.9600
C5—C6	1.385 (2)	C17—H17C	0.9600
C6—C7	1.389 (2)		
			
O1^i^—Mn1—O1	180.000 (1)	C6—C7—C2	120.84 (15)
O1—Mn1—O4	90.55 (5)	C6—C7—H7	119.6
O1^i^—Mn1—O4	89.45 (5)	N1—C8—C9	122.95 (16)
O1—Mn1—O4^i^	89.45 (5)	N1—C8—H8	118.5
O1^i^—Mn1—O4^i^	90.55 (5)	C9—C8—H8	118.5
O4—Mn1—O4^i^	180.00 (5)	C8—C9—H9	120.6
O1—Mn1—N1	92.44 (5)	C10—C9—C8	118.76 (15)
O1^i^—Mn1—N1	87.56 (5)	C10—C9—H9	120.6
O1—Mn1—N1^i^	87.56 (5)	C9—C10—C11	118.77 (15)
O1^i^—Mn1—N1^i^	92.44 (5)	C9—C10—H10	120.6
O4—Mn1—N1	87.02 (5)	C11—C10—H10	120.6
O4^i^—Mn1—N1	92.98 (5)	C10—C11—C13	123.27 (15)
O4—Mn1—N1^i^	92.98 (5)	C12—C11—C10	118.50 (15)
O4^i^—Mn1—N1^i^	87.02 (5)	C12—C11—C13	117.68 (14)
N1—Mn1—N1^i^	180.0	N1—C12—C11	123.06 (15)
C1—O1—Mn1	125.95 (11)	N1—C12—H12	118.5
Mn1—O4—H41	98 (2)	C11—C12—H12	118.5
Mn1—O4—H42	118 (2)	O3—C13—N2	121.63 (16)
H42—O4—H41	103 (3)	O3—C13—C11	117.65 (15)
C8—N1—Mn1	122.84 (11)	N2—C13—C11	120.70 (15)
C12—N1—Mn1	119.21 (11)	N2—C14—C15	113.64 (16)
C12—N1—C8	117.93 (15)	N2—C14—H14A	108.8
C13—N2—C14	117.30 (15)	N2—C14—H14B	108.8
C13—N2—C16	124.64 (15)	C15—C14—H14A	108.8
C16—N2—C14	118.06 (14)	C15—C14—H14B	108.8
O1—C1—C2	117.11 (15)	H14A—C14—H14B	107.7
O2—C1—O1	125.40 (15)	C14—C15—H15A	109.5
O2—C1—C2	117.49 (14)	C14—C15—H15B	109.5
C3—C2—C1	120.22 (15)	C14—C15—H15C	109.5
C7—C2—C1	120.33 (15)	H15A—C15—H15B	109.5
C7—C2—C3	119.45 (15)	H15A—C15—H15C	109.5
C2—C3—H3	119.8	H15B—C15—H15C	109.5
C4—C3—C2	120.44 (16)	N2—C16—C17	113.53 (16)
C4—C3—H3	119.8	N2—C16—H16A	108.9
C3—C4—H4	120.7	N2—C16—H16B	108.9
C5—C4—C3	118.55 (16)	C17—C16—H16A	108.9
C5—C4—H4	120.7	C17—C16—H16B	108.9
C4—C5—Br1	119.00 (13)	H16A—C16—H16B	107.7
C4—C5—C6	122.43 (16)	C16—C17—H17A	109.5
C6—C5—Br1	118.55 (13)	C16—C17—H17B	109.5
C5—C6—C7	118.23 (16)	C16—C17—H17C	109.5
C5—C6—H6	120.9	H17A—C17—H17B	109.5
C7—C6—H6	120.9	H17A—C17—H17C	109.5
C2—C7—H7	119.6	H17B—C17—H17C	109.5
			
O4—Mn1—O1—C1	−18.28 (14)	C14—N2—C16—C17	63.1 (2)
O4^i^—Mn1—O1—C1	161.72 (14)	O1—C1—C2—C3	176.80 (15)
N1—Mn1—O1—C1	−105.33 (14)	O1—C1—C2—C7	−3.0 (2)
N1^i^—Mn1—O1—C1	74.67 (14)	O2—C1—C2—C3	−3.0 (2)
O1—Mn1—N1—C8	−31.20 (13)	O2—C1—C2—C7	177.19 (14)
O1^i^—Mn1—N1—C8	148.80 (13)	C4—C3—C2—C1	178.07 (15)
O1—Mn1—N1—C12	147.52 (12)	C4—C3—C2—C7	−2.1 (2)
O1^i^—Mn1—N1—C12	−32.48 (12)	C3—C4—C5—Br1	−179.73 (13)
O4—Mn1—N1—C8	−121.63 (13)	C3—C4—C5—C6	1.7 (3)
O4^i^—Mn1—N1—C8	58.37 (13)	C5—C4—C3—C2	0.3 (3)
O4—Mn1—N1—C12	57.09 (13)	Br1—C5—C6—C7	179.61 (13)
O4^i^—Mn1—N1—C12	−122.91 (13)	C4—C5—C6—C7	−1.9 (3)
Mn1—O1—C1—O2	33.9 (2)	C5—C6—C7—C2	−0.1 (2)
Mn1—O1—C1—C2	−145.88 (11)	C6—C7—C2—C1	−178.19 (15)
Mn1—N1—C8—C9	−179.79 (13)	C6—C7—C2—C3	2.0 (2)
C12—N1—C8—C9	1.5 (2)	N1—C8—C9—C10	0.1 (3)
Mn1—N1—C12—C11	179.07 (12)	C11—C10—C9—C8	−1.0 (3)
C8—N1—C12—C11	−2.1 (2)	C9—C10—C11—C12	0.4 (2)
C14—N2—C13—O3	−4.2 (2)	C9—C10—C11—C13	−170.84 (16)
C14—N2—C13—C11	174.13 (15)	C10—C11—C13—O3	117.15 (18)
C16—N2—C13—O3	175.47 (16)	C10—C11—C13—N2	−61.2 (2)
C16—N2—C13—C11	−6.2 (2)	C12—C11—C13—O3	−54.2 (2)
C13—N2—C14—C15	78.2 (2)	C12—C11—C13—N2	127.49 (17)
C16—N2—C14—C15	−101.50 (19)	N1—C12—C11—C10	1.2 (3)
C13—N2—C16—C17	−116.6 (2)	N1—C12—C11—C13	172.96 (15)

Symmetry codes: (i) −*x*−3, −*y*−2, −*z*+1.

### Hydrogen-bond geometry (Å, °)

**Table:**

*D*—H···*A*	*D*—H	H···*A*	*D*···*A*	*D*—H···*A*
O4—H41···O2	0.86 (3)	1.82 (3)	2.6606 (18)	165 (3)
O4—H42···O3^ii^	0.82 (2)	1.92 (3)	2.742 (2)	166 (3)
C6—H6···O2^iii^	0.93	2.30	3.168 (3)	155
C10—H10···O2^iv^	0.93	2.44	3.353 (2)	168

Symmetry codes: (ii) ; (iii) *x*+1, *y*, *z*; (iv) .
